# Sensing Properties of g-C_3_N_4_/Au Nanocomposite for Organic Vapor Detection

**DOI:** 10.3390/bios13030315

**Published:** 2023-02-24

**Authors:** Atefeh Nasri, Babak Jaleh, Milad Daneshnazar, Rajender S. Varma

**Affiliations:** 1 Department of Physics, Faculty of Science, Bu-Ali Sina University, Hamedan 65174, Iran; 2 Institute for Nanomaterials, Advanced Technologies and Innovation (CxI), Technical University of Liberec (TUL), Studentska 1402/2, 46117 Liberec, Czech Republic

**Keywords:** gas sensor, g-C_3_N_4_, Au nanoparticles, laser ablation, organic vapors

## Abstract

Alleviating the increasingly critical environmental pollution problems entails the sensing of volatile organic compounds (VOCs) as a hazardous factor for human health wherein the development of gas sensor platforms offers an efficient strategy to detect such noxious gases. Nanomaterials, particularly carbon-based nanocomposites, are desired sensing compounds for gas detection owing to their unique properties, namely a facile and affordable synthesis process, high surface area, great selectivity, and possibility of working at room temperature. To achieve that objective, g-C_3_N_4_ (graphitic carbon nitride) was prepared from urea deploying simple heating. The ensuing porous nanosheets of g-C_3_N_4_ were utilized as a substrate for loading Au nanoparticles, which were synthesized by the laser ablation method. g-C_3_N_4_ presented a sensing sensitivity toward organic vapors, namely methanol, ethanol, and acetone vapor gases, which were significantly augmented in the presence of Au nanoparticles. Specifically, the as-prepared nanocomposite performed well with regard to the sensing of methanol vapor gas and offers a unique strategy and highly promising sensing compound for electronic and electrochemical applications.

## 1. Introduction

Exposure to different hazardous gases is extremely harmful for human health. Carbon monoxide, volatile organic compounds, nitrogen oxides, and hydrogen sulfide have been identified as the most dangerous gases. Monitoring hazardous gases in the air entails the utilization of high-efficiency sensors to identify and measure the gaseous and vaporous species [1]. Recently, gas sensors have been viewed admirably for the identification of harmful chemical vapors and toxic gases. Although solid state gas sensors are small in size and present affordable detectors with high sensitivity even in low concentrations of gases, their stability and limited measurement precision offer challenges. Consequently, it is an important endeavor to design a gas sensor with high selectivity and sensitivity. The incorporation of nanomaterials provides a high surface area for the better adsorption of gas species and improves the sensing performance. Additionally, a quick response and recovery, operation at ambient temperature, excellent reversibility, and ultrahigh sensitivity at extremely low concentrations can be achieved with the deployment of nanostructures as sensing materials [2,3,4].

Graphitic carbon nitride (g-C_3_N_4_), a two-dimensional polymeric semiconductor, has a high specific surface area. This eco-friendly material comprises the earth-abundant elements of nitrogen and carbon in a graphite-like layered structure, in which the robust covalent linkage among atoms leads to higher thermal and chemical stability. g-C_3_N_4_ has a wide indirect bandgap of 2.7 eV, thus making it a significant gas sensing candidate. Additionally, g-C_3_N_4_ possesses high electrical conductivity due to its unique delocalized conjugated structure [5,6,7,8]. Bulk g-C_3_N_4_ can be produced from N-enriched starting materials, namely melamine, dicyandiamide, urea, and thiourea, through facile synthetic methods. Furthermore, it can be exfoliated to nanosheets of a few layers, leading to a high surface area for loading nanoparticles without agglomeration [9]; g-C_3_N_4_ nanosheets are endowed with outstanding optical and electrical properties. This metal-free semiconductor can also enhance charge transferring and reduce the charge recombination [10]. Not surprisingly, assorted compounds of g-C_3_N_4_ with metal oxides and noble metals such as Co_3_O_4_/g-C_3_N_4_ [11], CuO-ZnO/g-C_3_N_4_ [12], NiO/g-C_3_N_4_ [13], Pd-WO_3_/g-C_3_N_4_ [14], and Pt-ZnO/g-C_3_N_4_ [10] have been deployed in gas sensing.

Noble metals (Au, Pt, Ag, etc.) have garnered much attention in catalytic systems and act as sensitizers in the gas sensing process. However, they tend to undergo agglomeration due to their considerable surface energy, leading to an adverse impact on their sensitization efficiency [15]. Decorating semiconductors with noble metal gold nanoparticles (Au NPs) not only fine-tunes their amount of charge carriers, but also impacts on their catalytic activity [16]. The surface modification with Au NPs enhances the electrical conductivity owing to their unique electronic storage abilities and great electrical conductivity and offers an efficient option to enhance the gas sensitivity and selectivity, and reduce the operating temperature [17]. The size of the metal nanoparticles and their interaction with supports are influenced by the deployed synthesis method including chemical and physical methods for the generation of nanoparticles [18]. For chemical synthesis methods, a higher temperature and the requirement for toxic chemicals are not only hazardous for the environment but also render them unaffordable. In addition, the prepared nanoparticles tend to undergo agglomeration in view of their high surface energy and consequent need for stabilizing agents during the synthesis process [18,19]. Notwithstanding the chemical methods, laser ablation in liquid (LAL) is an important physical method that produces colloidal nanoparticles from bulk materials, i.e., using a top-down route. It is a facile approach to form nano-size particles in large amounts, in which the nanoparticles have significant stability without any added surfactant in a liquid environment [20,21]. Moreover, the nanoparticles’ features can be controlled through appropriate laser parameters and a liquid medium [22].

As mentioned earlier, g-C_3_N_4_ has been produced by the transformation of urea by heating and subsequent exfoliation to generate g-C_3_N_4_ nanosheets by thermal treatment at 550 °C. Au NPs prepared by the LAL method could be supported onto the g-C_3_N_4_ nanosheets (CNN/Au) via a simple physical mixing. The characteristic analysis showed that Au NPs with an average crystalline size of 21 nm were successfully loaded on g-C_3_N_4_. In addition, the optical bandgap of g-C_3_N_4_ was reduced after loading Au NPs. The CNN/Au nanocomposite was utilized in the detection of hazardous vapors of acetone, methanol, and ethanol wherein the as-fabricated nanocomposite exhibited enormous sensitivity to methanol even at low concentrations.

## 2. Experimental Section

### 2.1. Materials and Instruments

To synthesis g-C_3_N_4_ nanosheets, urea (CAS NO. 57-13-6) was purchased from Merck Chemical Co, Hohenbrunn, Germany. A fiber laser (RFLP30Q, China) with a 1064 nm wavelength and maximal output of 30 W power was utilized to which gold plate was subjected to generate Au NPs with the purity of 99.9%. The crystalline structure was investigated through X-ray diffraction (XRD, Italstructure, ADP200, Italy) in the 2θ range of 10–90° at the wavelength of 0.154 nm. The functional groups and chemical bonds of samples were identified by Fourier transform infrared (FTIR, Perkin Elmer, SPECTRUM-GX, USA) spectra. Atomic force microscopy (AFM, Iran, Nanosurf Ni) was utilized to obtain the thickness of CNN nanosheets and roughness of the samples. High resolution transmission electron microscopy (HRTEM, JEOL 2100, Tokyo, Japan) images were acquired to detect morphology of CNN and Au NPs. Energy-dispersive X-ray spectroscopy (EDS) and elemental mapping analyses (TESCAN-MIRAIII-SAMX, Czechia Republic) were performed to investigate the constitutive elements of nanocomposite The optical properties of specimens were evaluated via Ultraviolet–visible (UV-Vis, PG Instruments-T80, China) spectroscopy.

### 2.2. Synthesis of g-C_3_N_4_ Nanosheets/Au Nanocomposite

**Figure 1:**
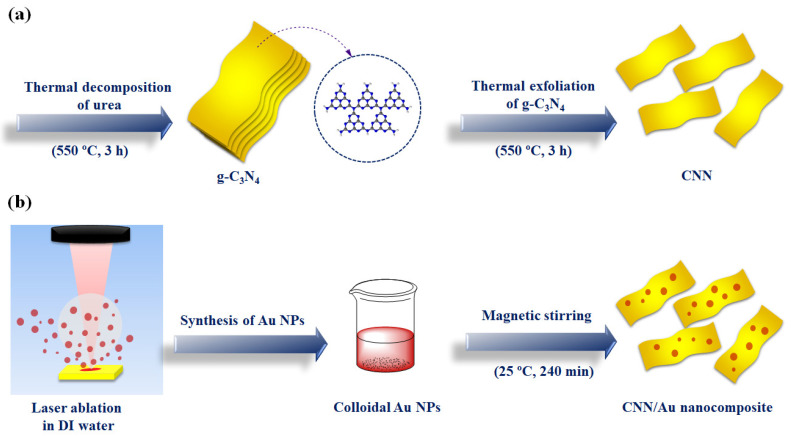
Representation for the synthesis of (**a**) CNN and (**b**) CNN/Au nanocomposite.

g-C_3_N_4_ was prepared by heating urea (20 g) in electric oven at 550 °C for 3 h as has been reported in our previous works [20,23]; about 1 g of produced yellowish material, g-C_3_N_4_ termed as CN. In order to exfoliate CN, the ensuing powder was ground with agate mortar and then was positioned in the crucible devoid of any cover and heated at 550 °C with heating speed of 3 °C/min for 3 h. As a result of this process, approximately 0.1 g of light milky powder was achieved, which was named as CNN (g-C_3_N_4_ nanosheets). Figure 1a illustrates a schematic for the preparation of g-C_3_N_4_ nanosheets.

To synthesize Au NPs, the LAL protocol was deployed by means of nanosecond fiber laser (RFLP30Q, 1064 nm, 30 W). At first, ultrasonic cleaning in acetone and deionized water (DW) media was utilized for the removal of foreign contaminants from surface of a metallic piece of Au (99.9%). It was then submerged in a glass vessel containing 10 mL of DW. The fiber laser with scanning speed of 200 mm/s, pulse length of 100 ns, and frequency of 20 kHz was deployed to irradiate the Au surfaces. To synthesize Au NPs, the beam of laser was fixated on the Au surface, in an area of 20 × 10 mm^2^. The Au NPs’ generation was easily recognized by the human eye when the color of the DW gradually changed to red. Stopping the laser irradiation of the Au target and refreshing the 10 mL of clean DW every 1 min prevented the formation of Au NP aggregates. The laser irradiation time to synthesize the colloidal Au NPs lasted nearly 3 min.

To synthesize CNN/Au nanocomposite, 0.1 g of CNN was scattered in ethanol–DW (2:1) solution using ultrasonic irradiation for 60 min and, subsequently, the colloidal Au NPs were added to it and scattered for 60 min again. After that, the suspension was stirred for 240 min and dried, as depicted in Figure 1b. All the processes were performed under ambient conditions.

### 2.3. Sensing Test

**Figure 2:**
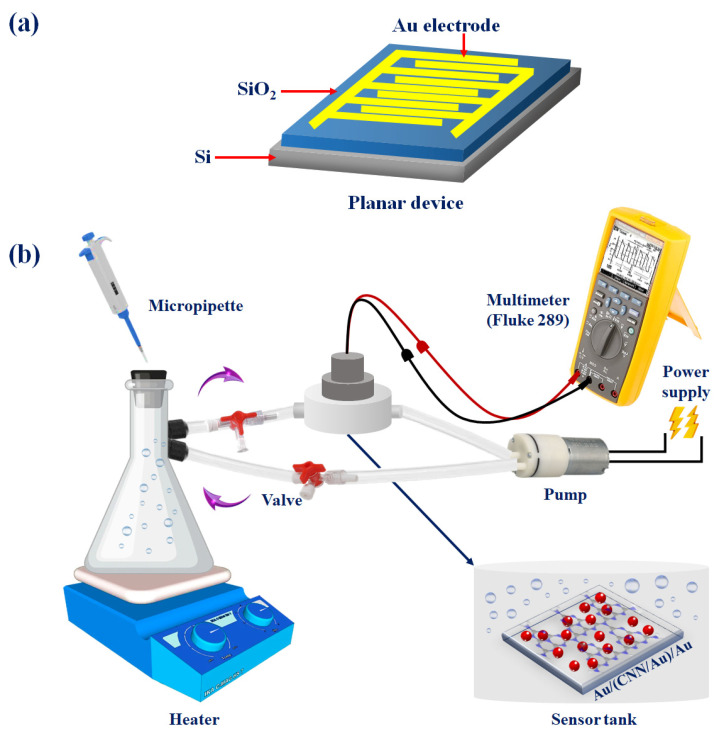
(**a**) Au/(CNN/Au)/Au planar devices and (**b**) illustrative representation for the vapor gas sensing evaluation arrangement.

As shown in Figure 2a, planar Au/(CNN)/Au and Au/(CNN/Au)/Au devices were used to evaluate the gas sensor. The electrodes were made up of an interdigital gold electrode that had a thickness of 30 µm and a width of 250 µm, as well as a distance of 250 µm among the electrode fingers and a SiO_2_/Si substrate using a CVD method and lithography [24,25]. The tests were performed for both substances, CNN and CNN/Au, for three types of organic vapors, ethanol, acetone, and methanol. As a first step, the desired nanomaterials were placed on a flat device by drop casting and allowed to dry at ambient temperature. For drop casting, 0.02 g of nanomaterials was ultrasonically dispersed in 5 mL of DW. After that, about 60 µL of the CNN and CNN/Au suspensions were dropped (2 times) on the surface of electrode. The planar device was placed inside the chamber and was connected to the two ends of the electrode via wires embedded inside the tank. Then, the output wires were connected to the two ends of the multimeter (Fluke 289) so that the resistance of the device was flat every moment (multimeter interval was set to 1 s to increase the accuracy of the test enough). As shown in Figure 2b, one end of the side of the chamber was connected to Erlen and the other end was linked to the pump. In this experiment, 1-L Erlenmeyer flask was used, and different volumes of acetone, methanol, and ethanol were inserted into the chamber using a syringe. Since the volume of chamber was one liter, each microliter refers to 1 ppm of ethanol, methanol, and acetone. Therefore, for 60 ppm, we needed to inject 60 microliters of that substance [26]. Since ethanol, methanol, and acetone vapors were deployed, a heater was used and the temperature regulated for each gas according to its boiling point (ethanol 79 °C, methanol 65 °C, and acetone 56 °C). The mini-DC pump played two roles here. The first one was related to accelerating the circulation of organic vapors, while the other one was to remove the remaining gases on the planer device as the testing method entailed injection of gas for 60 s and in 60 s air. This was measured 4 to 5 times for different concentrations, as the multimeter measured the resistance every second. The gas response was obtained through the following equation

$$\frac{\left|R_{a}-R_{g}\right|}{R_{a}}$$

where R_a_ is the resistance of the materials on the planar device in air and R_g_ is the resistance of the materials on the planar device to the gas. Response time is defined as the time required for a sensor to reach 90% of total response of the signal such as resistance exposure to the target gas. Recovery time is defined as the time required for a sensor to return to 90% of the original baseline signal upon removal of the target gas [27].

## 3. Results and Discussion

### 3.1. Characterization

**Figure 3:**
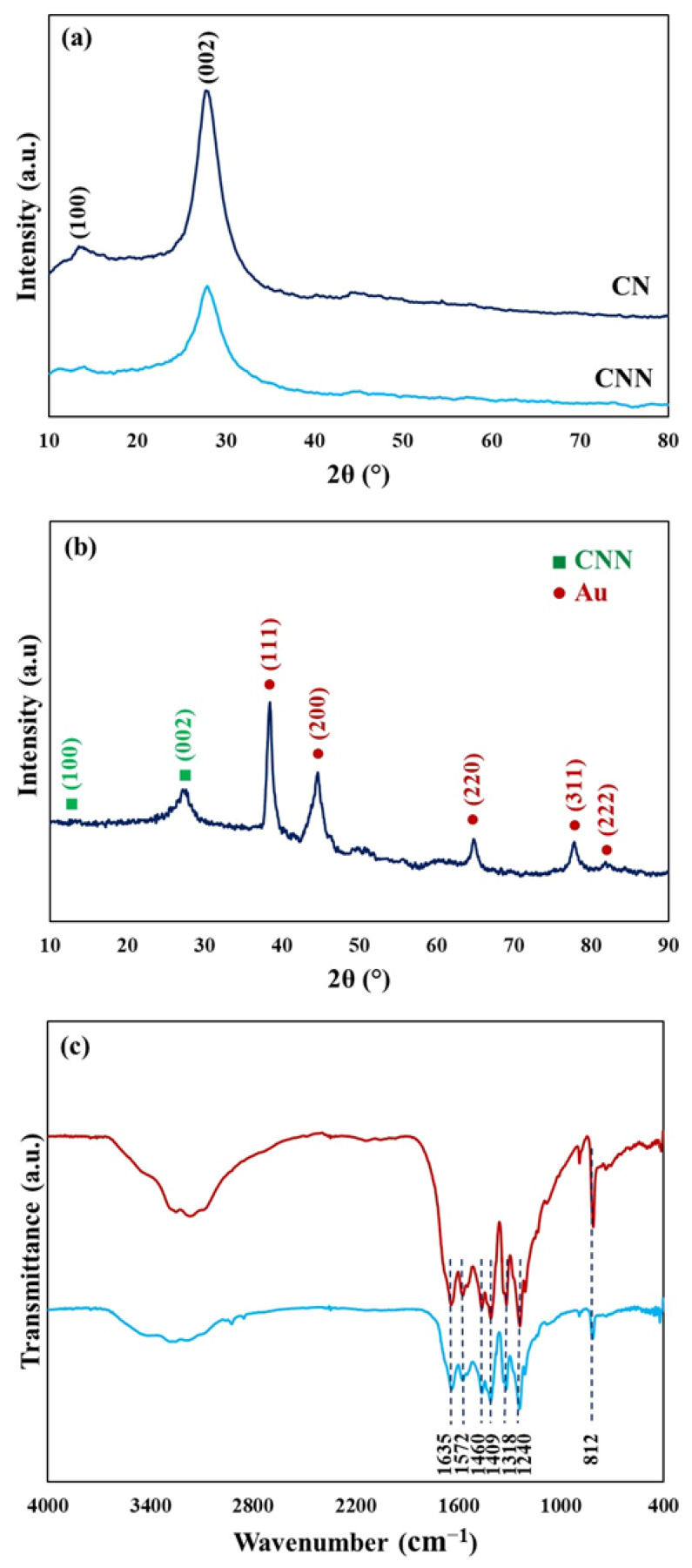
XRD patterns for (**a**) CN and CNN, (**b**) CNN/Au nanocomposite, and (**c**) FTIR spectra of CNN and CNN/Au nanocomposite.

To investigate the structure of the CNN/Au nanocomposite, the XRD analysis was applied. The XRD pattern of CN (Figure 3a) presents two characteristic diffraction peaks of g-C_3_N_4_ (JCPDS 87-1526), confirming the synthesis of CN. Two peaks at 13° (100) and 27.7° (002) are assigned to the inter-planer packaging of the heptazine system and the regular graphite-like interlayer stacking, respectively [23]. According to the XRD pattern of CN and CNN, it can be perceived that g-C_3_N_4_ nanosheets were successfully prepared, by reason of the significant reduction in (002) peak’s intensity subsequent to the exfoliation process. In Figure 3b, diffraction peaks at 38.4°, 44.6°, 64.7°, and 77.7° were observed, indicating the existence of Au NPs in the CNN/Au nanocomposite (JCPDS 00-004-0784) [28]. Average crystallite sizes of the Au NPs were obtained of about 21 nm by using Scherrer’s equation [29].

To identify the chemical bonds and functional groups of CNN and the CNN/Au nanocomposite, FTIR analysis was employed (Figure 3c). A broad peak between 3500–3000 cm^−1^ is allocated to N-H stretching [30]. Peaks located in the wavenumber range of 1700–1200 cm^−1^ correspond to C=N, C-N on the heterocyclic ring, and the C-N stretching vibration outside the ring [31]. A peak that appeared at 1460 cm^−1^ is attributed to a network triazine ring system linked with NH end groups [32]. The absorbance band at 812 cm^−1^ indicate vibration of the s-triazine ring [33], while the bands of CNN/Au are similar to the CNN spectrum, suggesting that the CNN structure does not change by loading Au NPs.

**Figure 4:**
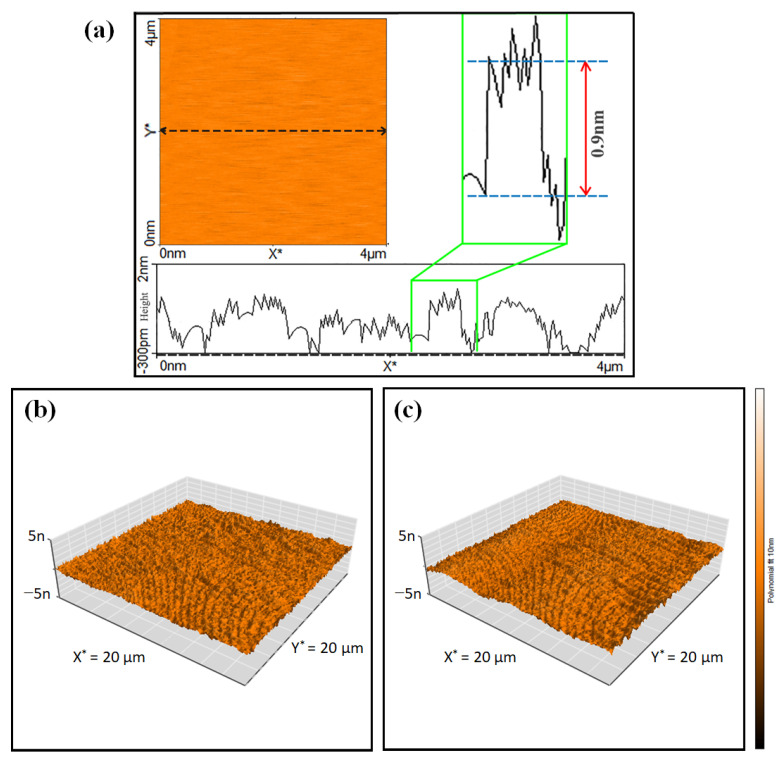
Two-dimensional AFM image and thickness profile of CNN (**a**) and 3D AFM images of CNN (**b**) and CNN/Au (**c**) samples.

AFM was employed to investigate the thickness of the CNN sample. Figure 4a illustrates that CNN has an average thickness of 0.9 nm, manifesting the successful exfoliation of CN into few-layered structures (CNN). According to the 3D AFM image (as shown in Figure 4b,c), values of the estimated average area roughness (S_a_) and root mean square roughness (S_q_) of CNN were changed from 186 pm and 234 pm, to 211 pm and 267 pm, respectively, in the presence of Au NPs. Therefore, Au NPs caused an increase in the surface roughness.

**Figure 5:**
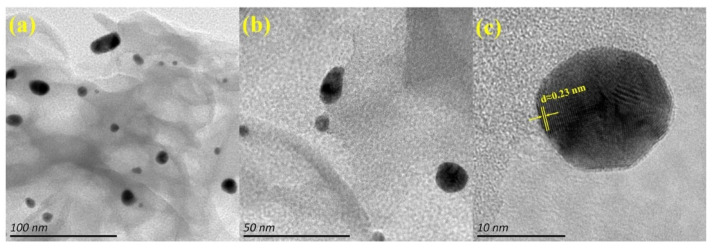
HRTEM images of CNN/Au nanocomposite with different magnitudes of 100 nm (**a**), 50 nm (**b**), and 10 nm (**c**).

Figure 5 shows the HRTEM images of the CNN/Au nanocomposite wherein the g-C_3_N_4_ nanosheets have a sheet-like shape with a smooth surface. The Au NPs with a black color have a spherical shape and are agglomerated in some regions of the g-C_3_N_4_ nanosheets (gray color regions) due to their high surface energy. Figure 5c reveals lattice borders with crystal plane distances of 0.23 nm, attributed to the (111) plane of Au NPs.

**Figure 6:**
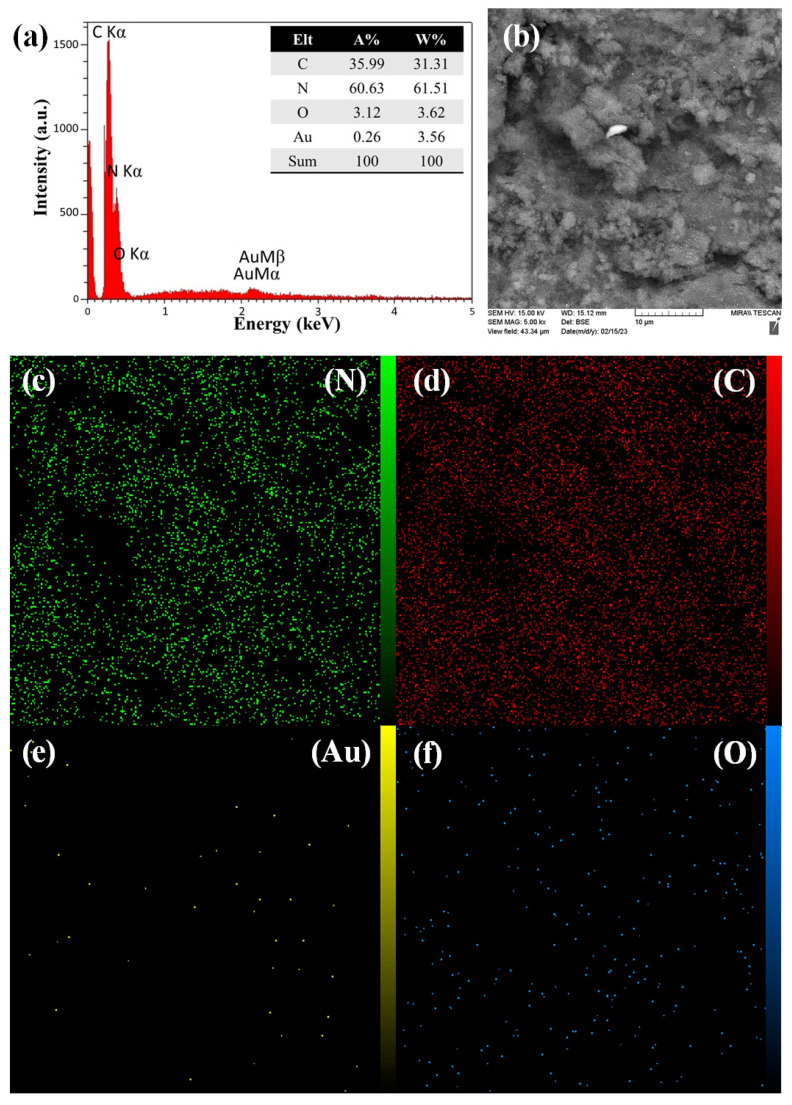
EDS pattern (**a**), representative EDS image (**b**), MAP of nitrogen element (**c**), MAP of carbon element (**d**), MAP of Au element (**e**), and MAP of oxygen element (**f**) of CNN/Au nanocomposite.

The corresponding EDS of the CNN/Au nanocomposite is depicted in Figure 6a, which confirms the presence of nitrogen, carbon, oxygen, and Au elements in the CNN/Au nanocomposite. The appearance of elemental Au in the EDS pattern is supported by the XRD results as well. Approximately, the uniform distribution of nitrogen, carbon, and oxygen on the CNN/Au nanomaterial and the low concentration of Au with non-uniform distribution were confirmed by MAP images, according to Figure 6b–f.

**Figure 7:**
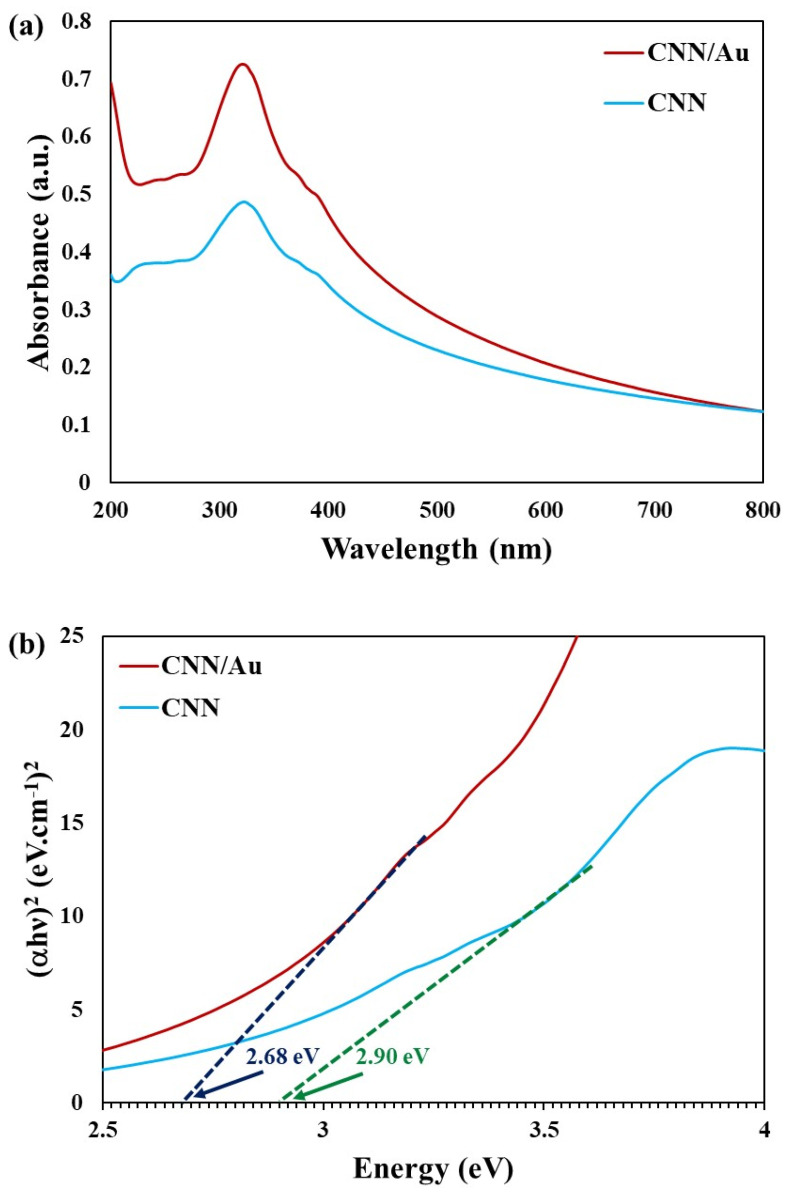
(**a**) UV-Vis spectra of CNN and CNN/Au compounds, (**b**) Tauc plot of inset UV-Vis spectra for CNN and CNN/Au.

The optical band gap of the semiconductors indicates the required energy of excitation and the transition of an electron from the valence to the conduction band. UV-Vis spectroscopy corresponds to the electron transition between the energy levels and for CNN and the CNN/Au nanomaterials, the spectra are shown in Figure 7a. As shown, two absorption peaks were located at about 325 and 400 nm, and can be assigned to the transition of π→π* and n→π*, respectively [34].

The Tauc equation can be utilized to calculate the optical band gap of CNN and the CNN/Au nanomaterials and is described by the following:

(αhν)^r^ = E_D_(hν − E_g_) (1)

where α, hν, E_g_, and E_D_ are the optical absorption coefficient, photon energy, optical band gap, and a constant, respectively. The r pertained to the nature of the electron transition and can be 2 or 0.5, corresponding to the direct or indirect transition band openings, respectively. The optical band gap energy was achieved by generalizing the linear part of the (αhν)^r^ vs hν plot to zero optical absorption [35,36]. The values of 2.9 and 2.68 eV were suggested for the optical direct band gap of CNN and CNN/Au nanocomposite, respectively (Figure 7b). Accordingly, the attendance of the metallic Au NPs affected the band gap reduction of the CNN/Au nanocomposite.

### 3.2. Gas Sensing Operation

**Figure 8:**
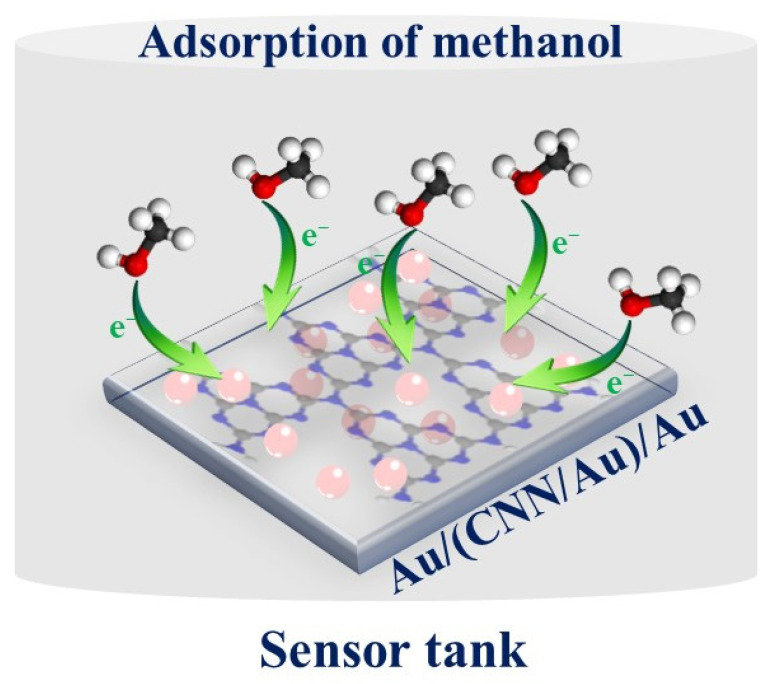
Schematic presentation of the methanol-sensing mechanism.

The sensing tests were initiated with an injection of gas for 60 s in the sensor tank using the mini-DC pump. After that, the sensor recovered its original state once fresh air was passed into the sensor tank. According to Figure 8, gas molecules (for example methanol molecules) can adsorb on the surface of the sensor by free electrons of oxygen and desorb when air is passed to recover the sensor.

**Figure 9:**
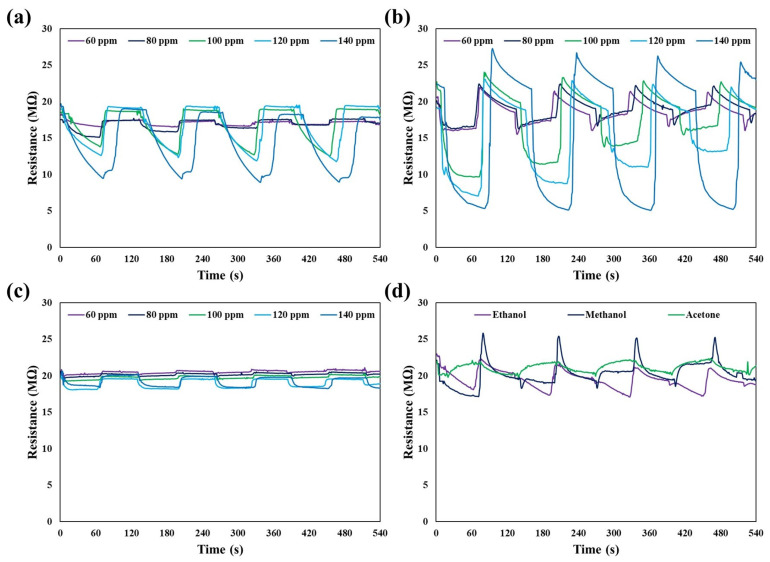
Responses of CNN and CNN/Au to gas upon exposure in four cycles from 60 ppm to 140 ppm of (**a**) ethanol, (**b**) methanol, (**c**) acetone, and (**d**) CNN in four cycles 140 ppm of ethanol, methanol, and acetone at 20 °C.

As shown in Figure 9, CNN and CNN/Au nanocomposites have different gas sensing characteristics for ethanol, acetone, and methanol at 60 ppm, 80 ppm, 100 ppm, 120 ppm, and 140 ppm concentrations. Here, the sensory properties of carbon nitride without gold were investigated only at a concentration of 140 ppm (because no response was observed at lower concentrations), and carbon nitride with loaded Au NPs at five different concentrations of 60 ppm, 80 ppm, 100 ppm, 120 ppm, and 140 ppm were measured. It was found that the sensing sensitivity of CNN was significantly improved in the presence of Au NPs and the maximum sensitivity was attained for CNN/Au towards 140 ppm of methanol vapor. Environmental factors can influence the sensor measurement such as temperature, humidity, and environmental noises, and as shown in Figure 9, a very small fluctuation was observed. Moreover, CNN is a semiconducting material that can be affected by temperature, so in each step of the sensor test, we tried to keep the temperature constant at 20 °C due to a slight difference in the results obtained by changing the temperature. The sensor response can be influenced by humidity as well. In order to avoid changing the humidity of the environment, we conducted all the tests on the same day.

Three significant parameters are considered here, which are individually called the maximum response, response time, and recovery time. As it appears in Table 1, the maximum response at 60 ppm for ethanol, acetone, and methanol is 1.1%, 3.2%, and 13.8%, in that order. By enhancing the concentration of the organic vapor, these values are increased. At a concentration of 140 ppm, methanol has the highest response at 72.6%, which was about 1.5 times more than ethanol and about 7 times more than acetone; the response to CNN reached 17.2%. In addition, the fastest response time and recovery time were obtained for ethanol and methanol, respectively. For more accuracy, four to five cycles were measured for each vapor, which shows the very good reproducibility of the sensor. Sensing tests were performed over four to five cycles because the volume of the gases entered were very low and the response decreased a bit after the fifth cycle. One of the reasons why methanol has a better reaction than acetone and ethanol can be found in the bond energy [37,38]. This is because the polarity of methanol is more than that of acetone and ethanol, which probably leads to a stronger interaction between methanol and CNN [9]. The fluctuations observed in the graph are because every second the resistance is measured, the system does not have enough time to stabilize and this causes oscillation. Finally, as shown in Table 1, the CNN/Au response at 140 ppm concentration was approximately 1.2, 5.7, and 4.22 times that of carbon nitride without Au, for acetone, ethanol, and methanol, in that order. In addition, two parameters of recovery time and response time are was summarized in Table 2 and Table 3, respectively.

A pore structure and high surface area in g-C_3_N_4_ offer additional active sites, causing fast gas adsorption/desorption [39,40]. The Au NPs not only increase the electrical conductivity of the CNN semiconductor, but can also increase oxygen species on the CNN substrate due to the spillover effect [39]. Therefore, when the Au NPs are loaded on the CNN substrate, the conductivity is increased, thus enhancing the response of the CNN substrate. The addition of Au NPs to CNN surfaces increases the reactive oxygen species on the exterior. According to the spillover effect of Au NPs, oxygen molecules are rapidly adsorbed and desorbed on the CNN and converted to oxygen species by electrons. Additionally, Au NPs can enhance the interaction between oxygen species adsorbing on the exterior and gas molecules [40,41,42,43].The response of the CNN/Au nanocomposite is compared with other g-C_3_N_4_-based composites in Table 4.

## 4. Conclusions

In summary, the CNN/Au nanocomposite was produced using a facile physical blending method. In the first step, g-C_3_N_4_ nanosheets were synthesized from the thermal exfoliation of g-C_3_N_4_ at 550 °C for 3 h. Afterwards, the colloidal Au NPs were synthesized by the LAL technique and supported on the CNN. According to characterization results, spherical-shaped Au NPs were successfully decorated on the nanosheets of CNN. The optical band gap of CNN was reduced from 2.90 to 2.68 eV after loading with Au NPs. The application of CNN for organic vapors’ sensing did not show any response at low concentrations of ethanol, methanol, and acetone, while the CNN/Au nanocomposite indicated significant sensitivity and exceptional repeatability for the recognition of methanol vapor even at low concentrations, and the maximal response of 72.6% was acquired for 140 ppm of methanol. Therefore, the CNN/Au nanocomposite can be a capable material for designing vapor sensors. The composition of CNN/Au with metal oxide nanoparticles, namely iron oxide, TiO_2_, and ZnO, among others, can probably improve the activity of CNN/Au toward gas sensing, which could be investigated in future studies.

## Figures and Tables

**Table 1:** The maximum response of the CNN- and CNN/Au-based sensor for different ppm of acetone, methanol, and ethanol vapors.

Sample	Gas Volume (ppm)	Ethanol (%)	Methanol (%)	Acetone (%)
CNN/Au	60	3.2	13.8	1.1
	80	6.9	16.4	1.6
	100	27.3	25.1	1.8
	120	39.7	54.2	5.7
	140	45.5	72.6	7.4
CNN	140	7.9	17.2	6.2

**Table 2:** The recovery times of the CNN- and CNN/Au-based sensor for different ppm of acetone, methanol, and ethanol.

Sample	Gas Volume (ppm)	Ethanol (s)	Methanol (s)	Acetone (s)
CNN/Au	60	31	19	23
	80	27	17	20
	100	25	16	19
	120	22	17	21
	140	20	14	20
CNN	140	19	18	17

**Table 3:** The response moments of the CNN- and CNN/Au-centered sensor for different ppm of acetone, methanol, and ethanol.

Sample	Gas Volume (ppm)	Ethanol (s)	Methanol (s)	Acetone (s)
CNN/Au	60	24	28	22
	80	23	27	21
	100	19	24	22
	120	17	22	18
	140	14	20	18
CNN	140	20	21	22

**Table 4:** Comparison of CNN/Au nanocomposite response with other g-C_3_N_4_-based composites (R_0_ is initial resistance before applying steam; R_g_ is the resistance of the material after applying organic vapor; V_a_ and V_g_ are material voltage when applying air and material voltage after application of organic vapor, respectively).

Nanocomposite	Target Gas (ppm)	Operating Temperature (°C)	Response	Ref
ZnO/g-C_3_N_4_	Ethanol (500)	350	350.1 (R_a_/R_g_)	[44]
SnS_2_/g-C_3_N_4_	Ethanol (500)	300	360 (R_a_/R_g_)	[45]
SnO_2_/g-C_3_N_4_	Acetone (20)	380	11 (V_g_/V_a_)	[46]
SnO_2_/g-C_3_N_4_	Ethanol (500)	340	150 (R_a_/R_g_)	[47]
Au/g-C_3_N_4_	NO_2_ (300)	450	80% (R_a_ − R_0_)/R_a_ × 100)	[48]
Ag/g-C_3_N_4_	Ethanol (50)	250	49.2 (R_a_/R_g_)	[39]
NiO/g-C_3_N_4_	CO (500)	240	2.729 (R_g_/R_a_)	[49]
ZnO/g-C_3_N_4_	CH_4_ (1000)	320	11.9 (R_a_/R_g_)	[50]
g-C_3_N_4_/TiO_2_	CO_2_ (1500)	450	88% (R_g_ − R_a_/R_a_ × 100)	[51]
ZnO/g-C_3_N_4_	NO_2_ (7)	RT	44.8 (R_g_/R_a_)	[52]
SnS_2_/g-C_3_N_4_	NO_2_ (1)	RT	503% (R_g_ − R_a_/R_a_ × 100)	[53]
g-C_3_N_4_/CuO	Acetone (1000)	RT	143.7 (R_g_/R_a_)	[54]
CNN/Au	Methanol (140)	65	72.6% (R_a_ − R_g_/R_a_ × 100)	This work

## Data Availability

Not applicable.
